# Multi-laboratory proficiency assessment for molecular and serological diagnosis of classical and African swine fever in the Americas

**DOI:** 10.3389/fvets.2026.1874382

**Published:** 2026-09-23

**Authors:** Chukwunonso Onyilagha, Orie Hochman, Gabriel Brawerman, Kalhari Goonewardene, Aruna Ambagala

**Affiliations:** 1 National Centre for Foreign Animal Disease, Canadian Food Inspection Agency, Winnipeg, MB, Canada; 2 Department of Veterinary Pathology, Western College of Veterinary Medicine, University of Saskatchewan, Saskatoon, SK, Canada; 3 Faculty of Veterinary Medicine, University of Calgary, Calgary, AB, Canada; 4 Department of Medical Microbiology and Infectious Diseases, Max Rady College of Medicine, University of Manitoba, Winnipeg, MB, Canada

**Keywords:** African swine fever, classical swine fever, inter-laboratory comparison, molecular assay, proficiency testing, serological assay, swine viral diseases, veterinary diagnostics

## Abstract

African swine fever (ASF) and classical swine fever (CSF) are economically significant transboundary viral diseases of pigs and are reportable to the World Organization for Animal Health (WOAH) due to their severe impact on production and international trade. Both diseases present with similar clinical signs; therefore, they cannot be reliably distinguished without laboratory diagnosis. Given that diagnostic assay performance could vary between laboratories, proficiency testing (PT) exercises play a critical role in ensuring the quality and reliability of laboratory results and in helping laboratories to identify and address procedural issues. As one of the WOAH reference laboratories for ASF and CSF, the National Center for Foreign Animal Disease (NCFAD) organized its first PT exercise for ASF and CSF in 2024, targeting the Americas. A total of 16 international laboratories from 11 countries participated in the PT exercise, which focused on front-line molecular and serological diagnostic assays that are essential for laboratory detection of ASF and CSF. The 15-sample molecular panel contained inactivated ASF (6 samples) and CSF (7 samples) viruses, in addition to two negative samples for genome detection. The 20-sample serological panel comprised serum samples containing antibodies to ASF (10 samples) and CSF (5 samples) viruses. The remaining five serum samples contained no antibodies to ASF or CSF viruses. All laboratories correctly identified the ASF virus (ASFV) genome-positive samples; however, only 85.7% of the laboratories correctly identified the CSF virus (CSFV) genome-positive samples. While 80% of participating laboratories accurately identified the ASF-positive serum samples, only 35.7% accurately identified the CSF-positive serum samples. Overall, most laboratories demonstrated high diagnostic accuracy for the molecular detection of ASF and CSF; however, further improvements are needed in the serological detection of ASF and CSF. The PT exercise helped participating laboratories strengthen their testing performance and advance their efforts toward accreditation.

## Introduction

ASF and CSF are considered two of the most significant viral diseases affecting the global swine industry, causing devastating economic losses due to high mortality, trade restrictions, and control costs (1–3). ASF, a hemorrhagic fever with near 100% fatality in domestic pigs (4), is caused by ASF virus (ASFV), a large double-stranded DNA virus belonging to the family *Asfarviridae*. CSF, also a hemorrhagic fever characterized by systemic hemorrhages and severe immunosuppression (5), is caused by CSF virus (CSFV), a positive-sense single-stranded RNA virus within the family *Flaviviridae*. ASFV has a large, complex genome (~170–190 kb), which encodes more than 150 proteins (4), while CSFV has a smaller genome (~12.3 kb) that encodes both structural and nonstructural proteins essential for viral replication (6). Despite being different in their genetic compositions, ASFV and CSFV induce similar clinical signs in pigs, including high fever, hemorrhagic lesions, and high mortality, complicating clinical differential diagnosis (4, 7). Both viruses exhibit notable global spread, with ASFV expanding from Africa into Europe, Asia, and the Americas, severely impacting global pork production and trade, and CSFV remaining endemic in parts of Asia, Europe, and Latin America, prompting ongoing control efforts (7–12).

Laboratory-based molecular and serological assays are critical for accurate and rapid diagnosis of ASF and CSF (13, 14), which in turn supports the prevention of disease spread, facilitates safe trade, and aids in outbreak control. Several molecular and serological assays have been developed and commercialized for ASF and CSF detection, allowing diagnostic laboratories to select tests based on factors such as speed, cost, and available infrastructure.

Routine proficiency testing (PT) in diagnostic laboratories is a vital quality assurance tool for evaluating the performance of laboratory assays. It enhances diagnostic reliability by standardizing protocols, identifying gaps in laboratory capacity, and improving outbreak response strategies (13, 15–17). This is particularly important given the economic consequences and international trade implications of ASF and CSF outbreaks, which often lead to movement bans and culling measures. Participation in PT exercises promotes continuous improvement, reduces false-positive and false-negative results, and increases confidence in diagnostic outcomes. It also provides additional benefits, including supporting accreditation, guiding corrective actions, and evaluating new techniques and technologies. Overall, improvements achieved through PT exercises enhance global preparedness and response capabilities against these devastating swine diseases.

As a WOAH reference laboratory for ASF and CSF, the NCFAD conducted its first PT exercise in the Americas in 2024, focusing on the frontline diagnostic assays required for the initial detection and surveillance of ASF and CSF. Sixteen laboratories from 11 countries in the Americas (Dominican Republic, Guatemala, Costa Rica, Brazil, El Salvador, Panama, Chile, Nicaragua, Mexico, Peru, and Belize) took part in this inaugural exercise. The serology panel consisted of heat-treated, lyophilized serum samples, while the molecular panel contained heat-treated and gamma-irradiated cell culture-amplified ASFV and CSFV strains in molecular transport medium (MTM).

## Materials and methods

### Sample selection for molecular and serological panel preparation

ASFV and CSFV used in the molecular panel (Table 1) were propagated in porcine primary leukocytes (PPLs) and porcine kidney 15 (PK-15) cell line for ASFV and CSFV, respectively, as described previously (18, 19). All serum samples used in the PT exercise were obtained from pigs experimentally infected with various strains of ASFV, CSFV, Bovine viral diarrhea virus (BVDV) and border disease virus (BDV) at the NCFAD’s Biosafety Level 3 Agriculture (BSL3-Ag) animal facility (Table 2). Samples 4 and 17 in the serology panel were obtained from pigs vaccinated with the chimeric CP7_E2alf, in which the E2 coding sequences from the BVDV CP7 strain are replaced with the E2 of the CSFV Alfort187 strain (20).

**Table 1:** Molecular panel information.

ID	Virus	Genotype	Dilution	Ct value	Conclusion
Sample 1	^*1^ASFV Georgia 2007/1	II	1/10	**31.24**	**Positive for ASFV**
Sample 2	CSFV Kanagawa	3.4	Undiluted	*25.07*	*Positive for CSFV*
Sample 3	CSFV Diepholz	2.3	Undiluted	*25.14*	*Positive for CSFV*
Sample 4	^*3^CSFV Diepholz	2.3	1/10	*28.75*	*Positive for CSFV*
Sample 5	MTM in cell culture media	NA	Undiluted	-	Negative for ASFV & CSFV
Sample 6	^*4^CSFV Alfort/187	1.1	1/10	*27.28*	*Positive for CSFV*
Sample 7	^*5^ASFV Malawi	VIII	1/10	**32.73**	**Positive for ASFV**
Sample 8	^*2^CSFV Kanagawa	3.4	1/10	*28.65*	*Positive for CSFV*
Sample 9	ASFV Malawi	VIII	Undiluted	**29.2**	**Positive for ASFV**
Sample 10	^*4^CSFV Alfort/187	1.1	1/100	*30.85*	*Positive for CSFV*
Sample 11	^*6^ASFV Lillie	XX	1/10	**32.88**	**Positive for ASFV**
Sample 12	ASFV Georgia 2007/1	II	Undiluted	**27.49**	**Positive for ASFV**
Sample 13	ASFV Lillie	XX	Undiluted	**29.33**	**Positive for ASFV**
Sample 14	MTM	NA	Undiluted	-	Negative for ASFV & CSFV
Sample 15	CSFV Alfort/187	1.1	Undiluted	*23.89*	*Positive for CSFV*

The molecular panel was prepared from multiple strains of cell culture-amplified ASFV and CSFV and tested by real-time PCR assays for ASF [Tignon et al. (31)] and CSF [Hoffmann et al. (25)]. Cycle threshold (Ct) values ≤ 35.44, positive; between 35.45 and 39.99, suspicious; and ≥ 40.00, negative. Bold, ASF-positive; italic, CSF-positive. *^1^Serial dilution was prepared from Sample 12; ^*2^Serial dilution was prepared from Sample 2; ^*3^Serial dilution was prepared from Sample 3; ^*4^Serial dilutions were prepared from Sample 15. ^*5^Serial dilution was prepared from Sample 9; ^*6^Serial dilution was prepared from Sample 13.

**Table 2:** Serology panel information.

ID	Virus	DPI	Dilution	ASF ELISA Kit #1	ASF ELISA Kit #2	IPT	CSF ELISA	Conclusion
Sample 1	ASFV Georgia 2007/1. P110	16	1/20	16.63	8.26	Neg	−1.6	Neg for anti-ASFV & CSFV Abs
Sample 2	ASFV OURT88-3/Malta’78. P2	56–27-8	1/20	**95.65**	**73.59**	**Pos**	−1.05	**Pos for antibodies to ASFV**
Sample 3	ASFV OURT88-3/Malta’78. P1	56–27-8	1/20	**98.91**	**86.07**	**Pos**	−13.36	**Pos for antibodies to ASFV**
Sample 4	CSFV Suvaxyn. P10 (NPLA titer: 320)	31	1/20	2.9	4.52	Neg	*51.94*	*Pos for antibodies to CSFV*
Sample 5	ASFV GUS Vietnam/Georgia 2007/1. P3	DPC 21	1/20	**96.05**	**77.45**	**Pos**	−3.18	**Pos for antibodies to ASFV**
Sample 6	CSFV Kanagawa. P1 (NPLA titer: 1280)	52	1/20	3.92	5.55	Neg	*87.41*	*Pos for antibodies to CSFV*
Sample 7	Normal Pig Serum	NA	Undiluted	6.84	3.78	Neg	2.35	Neg for anti-ASFV & CSFV Abs
Sample 8	CSFV Parma. P19-S-5	26	1/20	0.53	1.64	Neg	−8.61	Neg for anti-ASFV & CSFV Abs
Sample 9	ASFV GUS Vietnam/Georgia 2007/. P4	DPC 21	1/20	**100.53**	**69.47**	**Pos**	−1.74	**Pos for antibodies to ASFV**
Sample 10	Hyperimmune serum CSFV. P-03-04. (NPLA titer: 5120)	NA	1/20	8.06	7.41	Neg	*94.02*	*Pos for antibodies to CSFV*
Sample 11	ASFV Malta’78. P56	18	1/20	**68.08**	**95.5**	**Pos**	0.17	**Pos for antibodies to ASFV**
Sample 12	CSFV Brazil. P5 (NPLA titer: 640)	34	1/20	7.59	5.88	Neg	*63.37*	*Pos for antibodies to CSFV*
Sample 13	ASFV Estonia/2014. P4	11	1/20	**71.97**	**56.63**	**Pos**	4.28	**Pos for antibodies to ASFV**
Sample 14	ASFV OURT88-3/Malta’78. P4	56–27-8	1/20	**97.54**	**90.36**	**Pos**	6.94	**Pos for antibodies to ASFV**
Sample 15	BDV I-121. P13 (NPLA titer: 640)	48–15	1/20	7.72	5.38	Neg	13.46	Neg for anti-ASFV & CSFV Abs
Sample 16	ASFV GUS Vietnam/Georgia 2007/. P2	DPC 21	1/20	**100.1**	**63.06**	**Pos**	3.81	**Pos for antibodies to ASFV**
Sample 17	CSFV Suvaxyn. P11 (NPLA titer: 80)	31	1/20	0.41	4.18	Neg	*40.08*	*Pos for antibodies to CSFV*
Sample 18	ASFV OURT88-3/Malta’78. P3	56–27-8	1/20	**98.31**	**78.17**	**Pos**	5.68	**Pos for antibodies to ASFV**
Sample 19	ASFV Malta’78. P51	18	1/20	**50.66**	**86.33**	**Pos**	2.61	**Pos for antibodies to ASFV**
Sample 20	BVDV NADL. Pig 26 (NPLA titer: 1280)	141	1/20	0.28	4.63	Neg	4.49	Neg for anti-ASFV & CSFV Abs

The serology panel was prepared from sera obtained from domestic pigs infected with multiple strains of ASFV, CSFV, BDV, or BVDV and tested with INGEZIM PPA COMPAC Kit (ASF ELISA kit #1), ID Screen ASF Indirect Kit (ASF ELISA kit #2), IPT, NPLA, and IDEXX CSFV Antibody Test kit (CSF ELISA). Result interpretation: X% ≥ 50%, positive; X% X < 40%, negative; 50% > x% ≥ 40%, suspicious (ASF ELISA kit #1). S/*p* ≥ 40%, positive; S/*p* ≤ 30%, negative; 30% < S/*p* < 40%, suspicious (ASF ELISA kit #2). X% ≤ 30%, negative; > 30 to <40%, suspicious; ≥ 40%, positive (CSF ELISA). Bold, ASF-positive; italic, CSF-positive; DPI, days post-inoculation; DPC, days post-challenge; Neg, negative; Pos, positive.

### Evaluation of PrimeStore™ MTM for ASFV and CSFV genome stability

Unlike lyophilized serum, which is generally stable at room temperature for short periods of time, typically lasting from several days to a few weeks, thereby making it suitable for transport at room temperature, viral nucleic acids, especially viral RNA, are not stable beyond 48 h (21) at room temperature, and, if shipped at room temperature, could result in false-negative results when tested by the participating laboratories. PrimeStore™ MTM (Longhorn, Bethesda, MD, USA) has been shown to inactivate and stabilize viral nucleic acids, including ASFV (22, 23). To assess ASFV and CSFV nucleic acid stability following treatment with PrimeStore™ MTM, two representative samples used in the preparation of the molecular PT panel containing ASFV Georgia 2007/1 or CSFV Alfort/187 were mixed with MTM 1:3 as recommended by the manufacturer, and incubated at room temperature or at 40 °C for up to 30 days. As controls, the undiluted representative samples were also incubated at room temperature or at 40 °C for up to 30 days. The samples were tested by real-time PCR on 3, 7, 14, and 30 days after incubation.

### Virus inactivation and safety testing

To inactivate ASFV and CSFV, all samples were subjected to heat treatment (serum samples at 56 °C for 2 h or virus samples at 70 °C for 2 h in a water bath) followed by gamma irradiation (2-5 MRad) with Gammacell 220 Excel (Nordion®, Ottawa, ON, Canada). To confirm complete inactivation of CSFV and ASFV in the samples, a portion of each treated sample was passaged twice in appropriate cell types (PPLs for ASFV and PK-15 cell line for CSFV) as described previously (18, 19). The second passage of each sample was subsequently subjected to real-time PCR assay (ASFV and CSFV) and immunoperoxidase staining as described before (24, 25), to ensure that the samples had no live virus left. All samples were negative for live ASFV and CSFV by virus isolation and immunoperoxidase staining, as well as for nucleic acids of foot-and-mouth disease, avian influenza, Newcastle disease, and vesicular stomatitis viruses using agent-specific real-time PCR assays (26–30).

### Preparation of molecular and serology panels

Following inactivation and safety testing, samples were diluted as shown in Tables 1, 2. The serum samples were then aliquoted (1 mL per sample) into 5 mL glass vials and lyophilized using Gamma 2–20 (Martin Christ, Osterode am Harz, Germany) freezer-dryer according to the manufacturer’s instructions. The inactivated virus samples were mixed 1:3 with MTM to provide additional safety and stability to the samples, and aliquoted (0.5 mL per vial) into 2 mL cryovials. The PT panels contained 15 inactivated virus samples in MTM and 20 lyophilized serum samples for molecular and serology panel, respectively (Tables 1, 2).

### Evaluation of the molecular and serology panels at the NCFAD

MagMAX™ Pathogen RNA/DNA Kit, together with the MagMAX Express-96 Magnetic Particle Processor (Life Technologies, Burlington, ON, Canada), was used for extracting total nucleic acids from the panel samples following the manufacturer’s recommended protocol. Detection was performed using ASFV-specific (31) and CSFV-specific (25) real-time PCR assays. The real-time PCR assays were carried out on a Bio-Rad CFX96 Touch™ system (Bio-Rad, Hercules, CA, USA) using the TaqMan™ Fast Virus 1-Step Master Mix (Life Technologies, Burlington, ON, Canada) as described previously (32). Cycle threshold (Ct) values ≤ 35.44 were interpreted as positive, values between 35.45 and 39.99 as suspicious, and values ≥ 40.00 as negative.

A representative serological panel was evaluated at the NCFAD using commercially available INgezim PPA COMPAC® blocking ELISA kit (Gold Standard Diagnostics, Madrid, Spain), ID. Screen® ASF Indirect ELISA kit (Innovative Diagnostics, Grabels, France), and IDEXX CSFV Ab Test kit (IDEXX Laboratories, Markham, ON, Canada) for antibody detection, adhering to the overnight sample incubation procedure (where applicable) following manufacturers’ protocol. ASF ELISA positive and negative results were also confirmed by confirmatory Indirect Immunoperoxidase Test (IPT) (24). The IPT plates and the reagents were obtained from the European Union Reference Laboratory for ASF (EURL-ASF), and the assay was performed as previously described (33). The serum samples containing antibodies to CSFV, BDV, and BVDV were confirmed by the NCFAD Neutralization Peroxidase-Linked Assay (NPLA) assay as described previously (19).

### Participation, panel distribution, and results reporting

An invitation to participate in the 2024 NCFAD PT exercise for ASF and CSF was sent to the regional and national reference laboratories, as well as local veterinary diagnostic laboratories, in the Americas. The invitation was accepted in writing by 16 laboratories representing 11 countries in the region, and the participants were requested to complete a panel request form and an “acceptance of biological material” document. A document with sample details was provided to each laboratory to support import permit applications. The PT panels were then shipped at room temperature, and the shipping delays were tracked to help explain any inconsistency in results. The laboratories were provided with panel completion instructions and given the same deadline for the return of their results, except for a few that required additional time. Each participating laboratory was assigned a code number to maintain anonymity. The participating laboratories include two regional reference laboratories (labs 1 and 3), six national reference laboratories (labs 8, 13, 14, 17, 19, and 20), six regional veterinary diagnostic laboratories (labs 2, 4, 5, 7, 10, and 12), one quality control laboratory (lab 6), and one food analysis laboratory (lab 9). Labs 1, 2, 3, 4, 5, 6, 7, 8, 9, 10, 12, 13, 14, 17, and 20 participated in the ASFV real-time PCR component of the molecular panel, while labs 1, 2, 3, 5, 6, 7, 8, 9, 10, 12, 13, 14, 17, and 20 participated in the CSFV real-time PCR component. For the serology panel, labs 2, 5, 7, 8, 10, 12, 14, 17, 19, and 20 participated in the ASF ELISA component, while labs 1, 2, 4, 5, 6, 7, 8, 9, 10, 12, 14, 17, 19, and 20 participated in the CSF ELISA component. The results were reviewed and classified at the NCFAD as either consistent or inconsistent with expected results. A suspicious or negative result for a positive panel sample was classified as inconsistent. Individual and general reports were prepared and sent to each laboratory, followed by a virtual workshop to discuss the results, solutions to challenges, and answer questions from the participating laboratories.

### Data analysis

Figures for nucleic acid preservation by MTM, assay selection and nucleic acid extraction methods, and assay performance evaluation of participating laboratories were generated using GraphPad Prism version 9.3.1 for Windows (GraphPad Software, San Diego, CA, USA).

## Results

### MTM stabilizes viral nucleic acids for up to 30 days at 40 °C

Shipping panel samples at room temperature to participating laboratories helps to reduce shipping costs, especially for laboratories with a limited budget for PT programs. With MTM being able to preserve and stabilize samples, the NCFAD assessed the ability of MTM to help with sample preservation over a long period of time, mimicking the likely shipping duration for the panel samples.

For ASFV, results showed that the viral nucleic acid degradation (as indicated by the increasing Ct values) likely begins after 14 days (Figure 1A) at 40 °C in the absence of MTM. In contrast, MTM treatment preserved ASFV nucleic acid integrity for up to 30 days, with no noticeable degradation (Figure 1A). For CSFV, viral nucleic acid degradation was observed as early as 3 days, regardless of incubation temperature (Figure 1B). However, the addition of MTM stabilized the CSFV nucleic acid integrity for up to 30 days. Together, these results indicate that MTM treatment can preserve the panel samples for up to 30 days under extreme temperature conditions.

**Figure 1:**
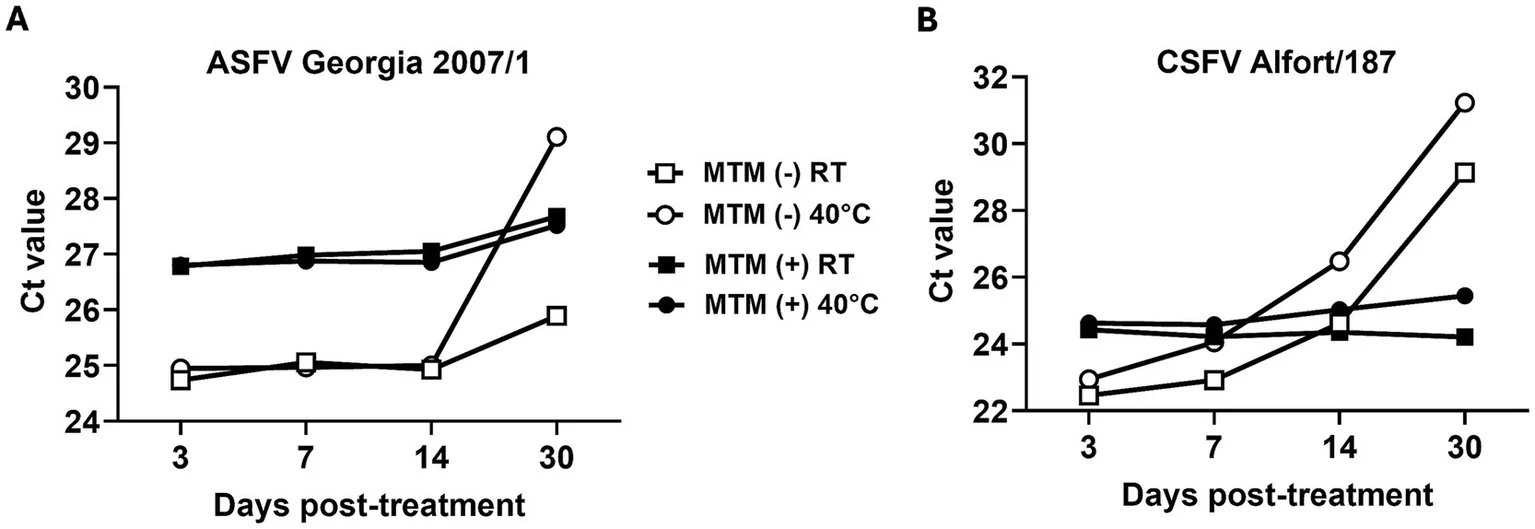
Nucleic acid preservation by MTM. Heat-inactivated and gamma-irradiated samples were treated with MTM and incubated at room temperature or 40 °C for 30 days. Samples were collected at 3, 7, 14, and 30 days and tested by real-time PCR for ASFV **(A)** and CSFV **(B)** to assess the Ct values.

### Characterization of the molecular and serology panels at the NCFAD

Following final preparation of the molecular and serology panels, representative panels were tested and analyzed at the NCFAD using molecular and serological assays for ASF and CSF to establish panel results prior to distribution. Results of the molecular panel showed six ASFV-positive and nine ASFV-negative samples, and seven CSFV-positive and eight CSFV-negative samples, with Ct values for positive samples ranging from 23.89 to 32.88 (Table 1). For the serology panels, NCFAD results for anti-ASFV antibodies showed 10 positive and 10 negative samples, while results for anti-CSFV antibodies showed five positive and 15 negative samples (Table 2). These ELISA results were confirmed by IPT and NPLA for ASF and CSF, respectively. The NCFAD results served as the reference results for assessing the performance of the participating laboratories.

### Molecular and serological assays used by participating laboratories

Several nucleic acid extraction methods and real-time PCR assays were utilized by the participating laboratories to generate their results. For ASFV nucleic acid extraction, six manual kits (Invitrogen PureLink Viral RNA/DNA Mini Kit; IDEXX RealPCR DNA/RNA Spin Column Kit; Invitrogen TRIzol Reagent; Roche High Pure PCR Template Preparation Kit; QIAGEN DNeasy Blood and Tissue Kits for DNA Isolation; QIAGEN QIAamp DNA Mini Kit) and six automated extraction-based kits (Applied Biosystems MagMAX Pathogen RNA/DNA Kit; Indical Bioscience IndiMag Pathogen IM48 Cartridge; undisclosed automated magnetic bead-based kit); Applied Biosystems MagMAX CORE Nucleic Acid Purification Kit; Zybio Nucleic Acid Extraction Kit; Promega Maxwell RSC Viral Total Nucleic Acid Purification Kit) were used (Figure 2A). For CSFV nucleic acid extraction, five manual kits (IDEXX RealPCR DNA/RNA Spin Column Kit; Invitrogen TRIzol Reagent; Roche High Pure PCR Template Preparation Kit; QIAGEN DNeasy Blood and Tissue Kits for DNA Isolation; Invitrogen PureLink Viral RNA/DNA Mini Kit) and six automated extraction-based kits (Applied Biosystems MagMAX Pathogen RNA/DNA Kit; Invitrogen MagMAX-96 Total RNA Isolation Kit; Applied Biosystems MagMAX CORE Nucleic Acid Purification Kit; undisclosed magnetic bead kit; Zybio Nucleic Acid Extraction Kit; Promega Maxwell RSC Viral Total Nucleic Acid Purification Kit) were used (Figure 2B). Overall, 50% of laboratories used manual extraction methods and 50% used automated magnetic bead-based methods for ASFV detection (Figure 2C). For CSFV detection, 40% of laboratories used manual extraction methods and 60% used automated magnetic bead-based methods (Figure 2D). These results highlight variability in laboratory capacity and workflow preferences among participating laboratories.

**Figure 2:**
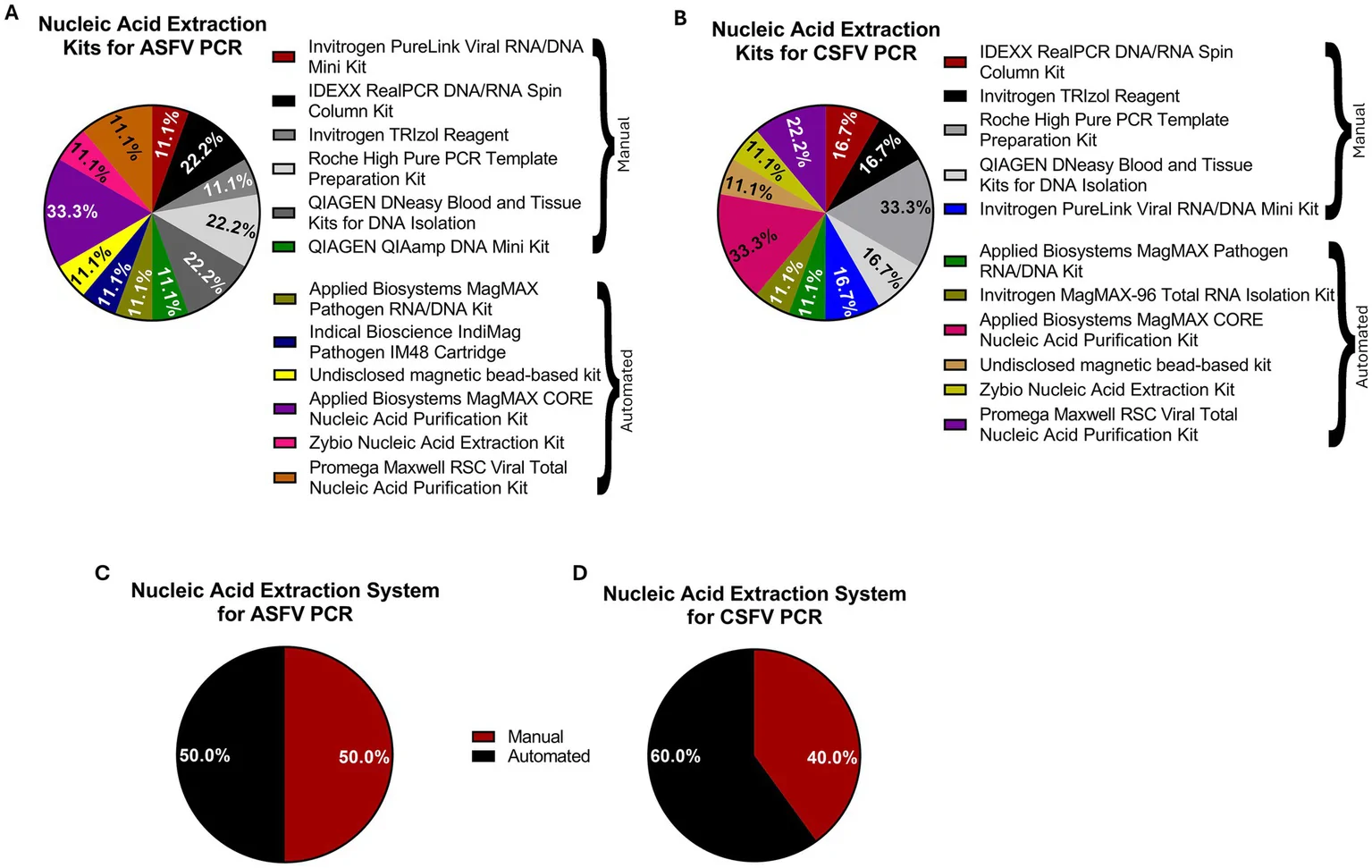
Nucleic acid extraction kits and systems. The nucleic acid extraction kits used for ASFV **(A)** and CSFV **(B)** real-time PCR assays, as well as the extraction systems used for ASFV **(C)** and CSFV **(D)** by participating laboratories, were compiled and presented as pie charts.

For molecular detection of ASFV, five ASFV-specific in-house real-time PCR assays [(31, 34, 35, 36); undisclosed assay from the WOAH Terrestrial Manual for ASF] and three different commercially available real-time PCR kits (VetMAX™ ASFV Detection Kit 2.0 from ThermoFisher, ID Gene™ ASF Duplex from Innovative Diagnostics, and RealPCR ASFV Mix from IDEXX) were used by the laboratories (Figure 3A). For CSFV molecular detection, four in-house real-time PCR assays [(25, 37); NVSL, and undisclosed assay from the WOAH Terrestrial Manual for CSF] and three different commercial ready-to-use real-time PCR kits (RealPCR CSFV Mix from IDEXX, VetMax™ Classical Swine Fever PCR kit from ThermoFisher, and virotype CSFV 2.0 RT-PCR Kit from INDICAL BIOSCIENCE) were used (Figure 3B).

For ASF serology, laboratories used three different commercial ELISA kits (ID Screen ASF Indirect, ID Screen ASF Competition, and INGEZIM PPA Compac; Figure 3C). CSF serology involved the use of three different ELISA kits (ID Screen CSF E2 Competition, IDEXX CSFV Ab Test, and RingBio CSF Antibody ELISA kit; Figure 3D).

This diversity in molecular and serological testing platforms likely reflects differences in resource availability, laboratory capacity, established workflows, or national regulatory approvals for specific kits and testing platforms.

### Performance evaluation of ASFV and CSFV real-time PCR across participating laboratories

For the ASFV real-time PCR (Table 3), the results were highly consistent, with 100% of the participating laboratories accurately identifying all 15 samples in the panel. These results demonstrate excellent proficiency in the molecular detection of ASFV, indicating that laboratories are utilizing their real-time PCR protocols effectively. However, the performance of the CSFV real-time PCR (Table 4) showed slight variability. While 85.7% of the laboratories correctly identified all 15 samples, 14.3% failed to accurately identify between 1 and 3 of the 15 panel samples.

**Table 3:** Evaluation of ASFV real-time PCR across participating laboratories.

Sample	NCFAD	Lab 1	Lab 2	Lab 3	Lab 4	Lab 5	Lab 6	Lab 7	*Lab 8		Lab 9	Lab 10	Lab 12	Lab 13	Lab 14	*Lab 17		*Lab 20	
S01	31.24	30.46	24.87	37.05	26.99	34.25	30.45	29.50	34.60	32.11	30.67	29.26	32.60	33.41	30.70	27.16	27.44	27.48	28.72
S02	-	-	-	-	-	-	-	-	-	-	-	-	-	-	-	-	-	-	-
S03	-	-	-	-	-	-	-	-	-	-	-	-	-	-	-	-	-	-	-
S04	-	-	-	-	-	-	-	-	-	-	-	-	-	-	-	-	-	-	-
S05	-	-	-	-	-	-	-	-	-	-	-	-	-	-	-	-	-	-	-
S06	-	-	-	-	-	-	-	-	-	-	-	-	-	-	-	-	-	-	-
S07	32.73	31.94	25.75	35.60	28.15	36.02	31.46	35.40	40.46	34.76	32.28	30.50	33.78	35.18	31.20	28.47	28.70	28.09	30.21
S08	-	-	-	-	-	-	-	-	-	-	-	-	-	-	-	-	-	-	-
S09	29.20	28.71	22.37	32.70	25.40	30.22	28.11	32.10	36.90	29.09	27.91	27.05	30.16	31.94	27.90	25.38	25.34	25.19	26.86
S10	-	-	-	-	-	-	-	-	-	-	-	-	-	-	-	-	-	-	-
S11	32.88	32.55	26.40	38.50	28.56	34.46	31.78	32.50	35.90	33.55	32.70	30.81	34.11	35.31	32.10	28.87	28.77	28.83	31.24
S12	27.49	26.68	21.40	32.20	24.00	28.08	27.05	28.60	29.30	27.89	26.78	25.62	28.32	30.15	27.20	24.06	24.27	24.81	27.69
S13	29.33	29.13	22.97	34.30	25.37	30.36	28.59	29.30	32.90	29.90	29.25	27.70	30.92	32.41	28.70	26.39	25.37	26.02	28.03
S14	-	-	-	-	-	-	-	-	-	-	-	-	-	-	-	-	-	-	-
S15	-	-	-	-	-	-	-	-	-	-	-	-	-	-	-	-	-	-	-
Assay/kit	Tignon et al. (31)	Tignon et al. (31)	IDEXX RealPCR ASFV Mix	Zsak et al. (34)	ID.vet ID Gene ASF Duplex	King et al. (35)	IDEXX RealPCR ASFV Mix	WOAH (undisclosed)	Fernández-Pinero et al. (36)	King et al. (35)	VetMAX ASFV Detection Kit 2.0	VetMAX ASFV Detection Kit 2.0	King et al. (35)	WOAH (undisclosed)	IDEXX RealPCR ASFV Mix	Zsak et al. (34)	Zsak et al. (34) (multiplex)	ID.vet ID Gene ASF Duplex	Zsak et al. (34)
Pos. Cutoff	≤35.44	≤35.99	≤35	<40	NR	<40	≤38	≤37	NR	NR	<45	<45	≤37	<40	<38	<40	<40	<40	≤37
Extraction method/kit	Automated (MagMAX Pathogen RNA/DNA Kit)	Manual (PureLink Viral RNA/DNA Mini Kit)	Manual (RealPCR DNA/RNA Spin Column Kit)	Automated (MagMAX Pathogen RNA/DNA Kit)	Manual (RealPCR DNA/RNA Spin Column Kit)	Manual (TRIzol Reagent)	Manual (High Pure PCR Template Preparation Kit)	Automated (IndiMag Pathogen IM48 Cartridge)	Manual (DNeasy Blood and Tissue Kits for DNA Isolation)	Manual (DNeasy Blood and Tissue Kits for DNA Isolation)	Undisclosed automated magnetic bead-based kit	Automated (MagMAX CORE Nucleic Acid Purification Kit)	Manual (High Pure PCR Template Preparation Kit)	Automated (Zybio Nucleic Acid Extraction Kit)	Manual (QIAamp DNA Mini Kit)	Automated (MagMAX CORE Nucleic Acid Purification Kit)	Automated (MagMAX CORE Nucleic Acid Purification Kit)	Automated (Maxwell RSC Viral Total Nucleic Acid Purification Kit)	Automated (Maxwell RSC Viral Total Nucleic Acid Purification Kit)

The ASF real-time PCR assay-specific results (Ct values) submitted by 15 participating laboratories were tabulated for each sample, together with the reference NCFAD results. Green, positive reference result; White, consistent result; −, No amplification; NR, not reported; *, Submitted two sets of results.

**Table 4:** Evaluation of CSFV real-time PCR across participating laboratories.

Sample	NCFAD	Lab 1	Lab 2	Lab 3	Lab 5	Lab 6	Lab 7	Lab 8	Lab 9	Lab 10	Lab 12	Lab 13	Lab 14	Lab 17	*Lab 20	
S01	-	-	-	-	-	-	-	-	-	-	-	-	-	-	-	-
S02	25.07	23.62	19.95	37.55	28.30	30.30	25.40	30.30	-	22.91	24.16	34.31	25.00	32.29	26.45	31.85
S03	25.14	23.84	20.15	30.39	29.71	30.34	25.40	29.60	33.06	22.96	24.08	29.44	25.90	28.00	26.47	33.43
S04	28.75	27.05	23.18	34.22	32.77	33.93	28.90	31.50	35.11	26.39	27.89	32.06	28.70	31.10	29.39	31.09
S05	-	-	-	-	-	-	-	-	-	-	-	-	-	-	-	-
S06	27.28	25.31	21.92	36.60	30.09	32.24	27.30	32.20	35.74	24.90	27.42	31.74	27.20	33.04	28.83	32.80
S07	-	-	-	-	-	-	-	-	-	-	-	-	-	-	-	-
S08	28.65	26.80	23.21	41.99	30.51	33.87	28.70	34.00	-	26.48	28.78	37.29	29.50	35.34	32.10	31.52
S09	-	-	-	-	-	38.59	-	-	-	-	-	-	-	-	-	-
S10	30.85	28.69	25.00	40.54	33.58	36.68	30.70	36.80	-	28.22	30.58	36.99	30.40	37.43	33.43	34.84
S11	-	-	-	-	-	-	-	-	-	-	-	-	-	-	-	-
S12	-	-	32.65	-	-	-	-	-	-	-	-	-	-	-	-	-
S13	-	-	-	-	-	-	-	-	-	-	-	-	-	-	-	-
S14	-	-	-	-	-	-	-	-	-	-	-	-	-	-	-	-
S15	23.89	23.21	18.84	31.94	26.45	29.51	24.20	28.20	33.67	21.40	23.88	28.65	24.20	28.97	26.27	26.05
Assay/kit	Hoffmann et al. (25)	Hoffmann et al. (25)	IDEXX RealPCR CSFV Mix	Eberling et al. (37)	Hoffmann et al. (25)	IDEXX RealPCR CSFV Mix	Hoffmann et al. (25)	Hoffmann et al. (25)	VetMAX CSFV Reagent	Hoffmann et al. (25)	NVSL	WOAH (undisclosed)	Hoffmann et al. (25)	Eberling et al. (37)	Indical Bio virotype CSFV 2.0 RT-PCR	Hoffmann et al. (25)
Pos. Cutoff	≤35.44	≤35.99	≤35	NR	≤38	≤38	≤38	NR	<40	<35.99	35.72	<37.99	<40	<40	≤37	≤37
Extraction method/kit	Automated (MagMAX Pathogen RNA/DNA Kit)	Automated (MagMAX Pathogen RNA/DNA Kit)	Manual (RealPCR DNA/RNA Spin Column Kit)	Automated (MagMAX-96 Total RNA Isolation Kit)	Manual (TRIzol Reagent)	Manual (High Pure PCR Template Preparation Kit)	Automated (MagMAX CORE Nucleic Acid Purification Kit)	Manual (DNeasy Blood and Tissue Kits for DNA Isolation)	Undisclosed automated magnetic bead-based kit	Automated (MagMAX CORE Nucleic Acid Purification Kit)	Manual (High Pure PCR Template Preparation Kit)	Automated (Zybio Nucleic Acid Extraction Kit)	Manual (PureLink Viral RNA/DNA Mini Kit)	Automated (MagMAX CORE Nucleic Acid Purification Kit)	Automated (Maxwell RSC Viral Total Nucleic Acid Purification Kit)	Automated (Maxwell RSC Viral Total Nucleic Acid Purification Kit)

The CSFV real-time PCR assay-specific results (Ct values) submitted by 14 participating laboratories were tabulated for each sample, together with the reference NCFAD results. Green, positive reference result; White, consistent result; Yellow, inconsistent result; −, no amplification; NR, not reported; *, submitted two sets of results.

**Figure 3:**
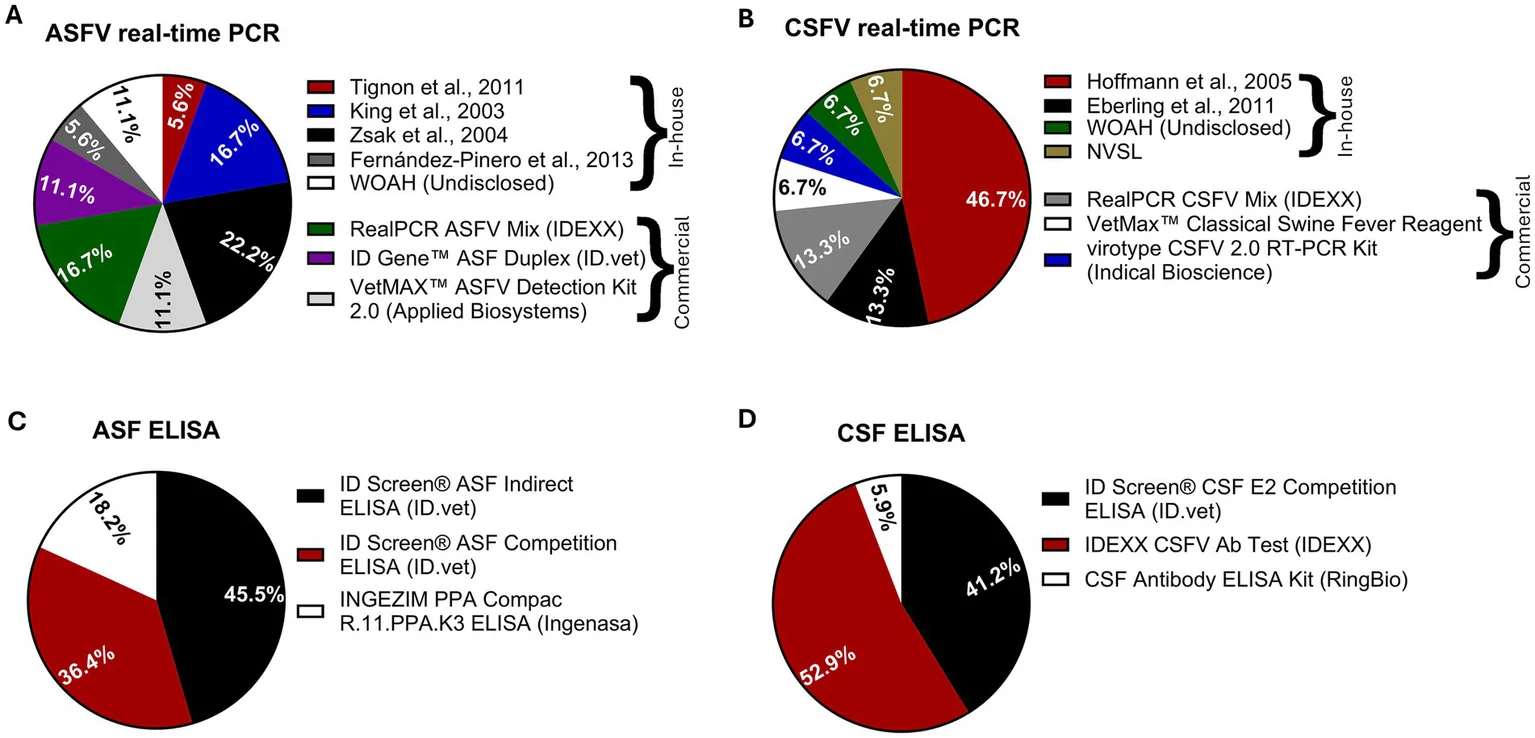
Molecular and serological assay selection. The real-time PCR assays for ASFV **(A)** and CSFV **(B)**, and the assays for ASF ELISA **(C)** and CSF ELISA **(D)** submitted by participating laboratories, were compiled and presented as pie charts.

Individual laboratory detection accuracy for ASFV and CSFV real-time PCR is shown in Figures 4A,B, respectively. Although most laboratories performed well, the discrepancies observed for CSFV suggest that some laboratories may benefit from further review of assay setup, template handling, or PCR cycling conditions to ensure optimal detection accuracy.

**Figure 4:**
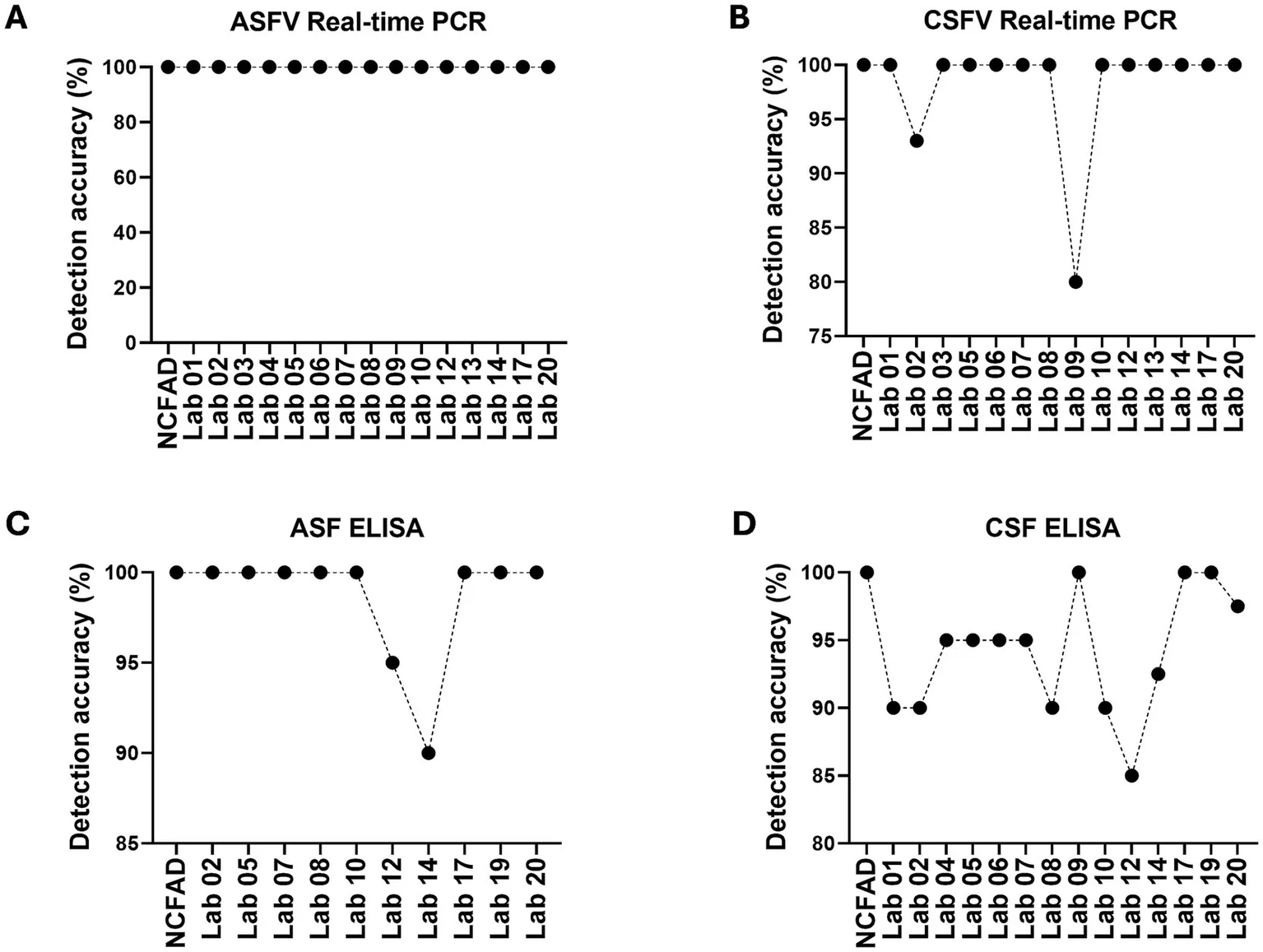
Detection accuracy of the participating laboratories for real-time PCR and ELISA. The real-time PCR and ELISA results submitted by participating laboratories were represented graphically to show the detection accuracy for ASFV real-time PCR **(A)**, CSFV real-time PCR **(B)**, ASF ELISA **(C)**, and CSF ELISA **(D)**.

### Performance evaluation of ASF and CSF ELISA across participating laboratories

For the ASF ELISA (Table 5), 80% of the participating laboratories produced results consistent with those obtained by NCFAD. The remaining 20% of laboratories failed to accurately identify 1–2 out of 20 samples in the serology panel. Although the number of inaccuracies was relatively small, these discrepancies still indicate methodological issues that could affect the reliability of ASF serological testing in some laboratories. In contrast, the overall performance for CSF ELISA (Table 6) was notably lower than that observed for ASF ELISA. Only 35.7% of laboratories obtained accurate results for all samples, while 64.3% had inconsistent results, misidentifying 1–3 out of the 20 panel samples. Individual laboratory detection accuracy is shown for ASFV ELISA (Figure 4C) and CSF ELISA (Figure 4D). The higher rate of discordant results in CSF ELISA highlights greater variability in protocols and assays among laboratories, suggesting that further optimization and harmonization of protocols is required.

**Table 5:** Evaluation of ASF ELISA across participating laboratories.

Sample	NCFAD	Lab 2	Lab 5	Lab 7	Lab 8	Lab 10	Lab 12	Lab 14	Lab 17	Lab 19	*Lab 20	
S01	16.63	15	71.52	31.35	11.15	98	8.123	2.027	4.181	87	87.17	19
S02	95.65	58	9.95	98.1	83.3	10	58.769	51.186	63.737	10	11.60	96
S03	98.91	70	7.34	98.84	90.26	9	80.184	66.71	81.996	10	8.51	99
S04	2.90	7	81.67	7.51	6.29	123	3.815	2.544	2.815	77	82.63	4
S05	96.05	62	13.88	99.01	84.69	15	72.43	69.383	73.89	14	34.07	94
S06	3.92	8	80.47	22.28	6.2	96	5.476	1.682	3.071	84	86.55	11
S07	6.84	8	83.95	28.96	6.42	81	7.692	1.509	2.559	98	89.54	16
S08	0.53	7	83.97	23.76	6.51	101	6.276	2.113	2.645	94	86.13	16
S09	100.53	57	13.65	99.92	70.1	14	59.384	33.161	53.583	12	24.69	99
S10	8.06	6	71.73	28.63	6.58	82	7.015	1.94	2.815	86	75.72	9
S11	68.08	74	12.13	82.59	91.41	13	99.446	82.061	93.941	21	15.93	66
S12	7.59	8	79.93	14.6	5.73	111	6.03	1.768	2.986	96	90.98	8
S13	71.97	41	31.58	73.76	53.27	37	38.338	27.038	79.778	35	33.14	65
S14	97.54	76	5.66	100	97.24	3	80.492	60.931	94.795	4	12.32	98
S15	7.72	7	87.14	22.69	5.23	82	8.061	1.596	2.815	98	88.30	12
S16	100.10	46	13.14	100.08	65.86	10	62.83	46.529	58.105	11	12.22	99
S17	0.41	7	74.54	28.22	4.29	94	7.261	2.027	3.071	91	78.92	8
S18	98.31	50	11.06	99.5	86.13	13	65.723	56.188	82.423	13	36.86	95
S19	50.66	58	12.72	58.91	75.79	13	67.569	63.346	57.337	18	15.72	64
S20	0.28	6	75.98	8.33	5.08	94	6.276	1.854	2.474	98	88.92	6
Assay/kit	INGEZIM PPA Compac	ID.vet ID Screen ASF Indirect	ID.vet ID Screen ASF Competition	INGEZIM PPA Compac	ID.vet ID Screen ASF Indirect	ID.vet ID Screen ASF Competition	ID.vet ID Screen ASF Indirect	ID.vet ID Screen ASF Indirect	ID.vet ID Screen ASF Indirect	ID.vet ID Screen ASF Competition	ID.vet ID Screen ASF Competition	INGEZIM PPA Compac
Pos. Cutoff	≥50	≥40	≤40	≥50	≥40	≤40	≥40	≥40	≥40	≤40	≤40	≥50

The results of the ASF ELISA submitted by 10 participating laboratories, based on assay-specific interpretations, were tabulated for each sample, together with the reference NCFAD results. Green, positive reference result; White, consistent result; ellow, inconsistent result; *, submitted two sets of results.

**Table 6:** Evaluation of CSF ELISA across participating laboratories.

Sample	NCFAD	Lab 1	Lab 2	Lab 4	Lab 5	Lab 6	*Lab 7		Lab 8	Lab 9	Lab 10	Lab 12	*Lab 14		Lab 17	Lab 19	*Lab 20	
S01	−1.60	−5	90	9.994	3.92	12.465	89.64	90.78	94.79	−2.242	95	15.64	86.926	4.88	4.27	6.44	87.166	−6.1
S02	−1.05	−2	94	8.112	−1.61	19.183	85.37	88.65	104.35	−4.6554	94	12.728	88.285	−2.99	8.217	3.149	61.251	−5.0
S03	−13.36	−3	99	−0.13	−2.27	10.942	89.38	90.71	97.98	−6.5565	92	13.311	92.039	−17.3	1.256	−6.44	94.652	−8.3
S04	51.94	30	66	41.337	47.27	51.177	54.55	36.16	58.16	52.768	64	29.284	61.23	41.7	53.211	49.532	19.407	45.3
S05	−3.18	1	93	−7.008	5.04	6.302	91.39	89.12	99.94	−9.9135	89	20.382	97.929	3.4	−2.045	5.362	61.186	−12.1
S06	87.41	71	19	74.043	78.56	78.393	21.03	7.43	21.69	88.432	22	41.181	22.33	77.9	83.567	84.057	5.545	81.7
S07	2.35	−2	88	−5.905	2.69	7.271	86.92	87.39	98.21	1.201	88	23.128	92.362	−40.4	6.566	−3.149	94.118	−11.3
S08	−8.61	6	91	−0.714	10.39	8.934	92.75	91.37	102.44	1.108	91	20.465	91.133	5.13	7.427	1.305	92.58	−11.6
S09	−1.74	4	91	0.973	9.77	7.618	90.42	91.11	98.87	−0.3795	90	25.623	86.019	−119	7.643	3.064	60.284	−13.0
S10	94.02	88	6	87.151	89.24	87.535	7.12	3.12	5.91	92.463	7	55.657	6.926	44.9	91.46	90.61	3.417	89.1
S11	0.17	−8	99	6.424	−2.27	11.704	89.77	92.9	98.19	−6.747	94	0.249	91.456	−6.44	3.911	−2.582	63.056	−13.6
S12	63.37	41	40	48.021	60.3	63.573	39.48	21.57	43.85	61.014	40	35.44	40.583	52.6	62.899	58.553	15.667	55.7
S13	4.28	−12	90	−12.265	5.39	3.186	93.59	91.97	100.09	−7.9005	89	3.826	99.029	−2.5	−7.93	5.362	61.896	−14.5
S14	6.94	−3	89	−11.421	3.37	22.368	91.45	87.59	102.64	−0.2235	89	13.56	95.469	−87.1	−0.395	−1.39	62.798	−3.4
S15	13.46	4	77	4.348	−0.03	14.266	84.01	70.74	89.99	5.487	82	16.056	91.974	7.5	4.055	5.674	72.527	2.1
S16	3.81	4	86	6.944	4.77	9.349	93.14	92.1	100.64	0.4425	91	16.057	93.01	4.88	3.265	6.78	60.155	−1.1
S17	40.08	26	65	14.337	35.66	33.38	73.52	45.06	72.41	44.0035	67	29.95	71.909	28.7	40.589	43.291	26.886	29.7
S18	5.68	−1	91	5.646	0.56	9.903	85.63	86.73	100.98	−2.08835	83	20.049	96.505	−3.49	2.476	2.922	61.702	−4.4
S19	2.61	−9	96	1.298	−6.04	12.604	82.65	90.91	102.61	−9.176	86	1.247	97.152	−93.9	−1.543	−1.674	63.572	−13.8
S20	4.49	−11	97	5.062	1.77	14.475	89.12	88.39	104.82	−13.602	85	13.56	97.346	−5.7	1.256	9.674	92.045	−9.5
Assay/kit	IDEXX CSFV Ab	IDEXX CSFV Ab	ID.vet ID Screen CSF E2 Competition	IDEXX CSFV Ab	IDEXX CSFV Ab	IDEXX CSFV Ab	ID.vet ID Screen CSF E2 Competition	ID.vet ID Screen CSF E2 Competition	ID.vet ID Screen CSF E2 Competition	IDEXX CSFV Ab	ID.vet ID Screen CSF E2 Competition	RingBio CSFV Antibody ELISA	ID.vet ID Screen CSF E2 Competition	IDEXX CSFV Ab	IDEXX CSFV Ab	IDEXX CSFV Ab	ID.vet ID Screen CSF E2 Competition	IDEXX CSFV Ab
Pos. Cutoff	≥40	≥40	≤50	≥40	≥40	≥40	≤50	≤50	≤50	≥40	≤50	≥40	≤50	≥40	≥40	≥40	≤50	≥40
Overnight incubation	Yes	No	No	No	No	No	No	Yes	No	Yes	No	No	No	No	Yes	Yes	Yes	Yes

The results of the CSF ELISA submitted by 14 participating laboratories, based on assay-specific interpretations, were tabulated for each sample, together with the reference NCFAD results. Green, positive reference result; white, consistent result; yellow, inconsistent result; *, submitted two sets of results.

## Discussion

The ASF and CSF proficiency testing exercise organized by the NCFAD successfully evaluated diagnostic performance across 16 laboratories from 11 countries, providing valuable insight into laboratory readiness for two economically devastating swine diseases. Overall, most of the participating laboratories demonstrated acceptable proficiency, especially in real-time PCR detection, with notable variability in ELISA performance. Strengthening diagnostic consistency is critical for early outbreak detection, rapid response, and minimizing trade disruptions associated with ASF and CSF.

A major finding of the PT exercise was the high level of consistency in real-time PCR detection, particularly for ASF. All laboratories achieved 100% accuracy in the ASFV real-time PCR panel, despite using eight different PCR protocols and a mix of manual and automated extraction methods. This aligns well with the high analytical sensitivity and specificity of ASFV real-time PCR protocols recommended by the WOAH for routine screening and outbreak detection (38). The performance for CSFV real-time PCR was slightly lower, with 14.3% of laboratories misidentifying 1–3 samples, likely due to reduced assay sensitivity, variability in sample handling or nucleic acid extraction systems. In addition, an increase of approximately 3 Ct values was expected for each serially diluted panel sample, and the results submitted by the laboratories were mostly consistent, with only a few showing slight inconsistencies. Nonetheless, overall real-time PCR proficiency was encouraging, showing that real-time PCR capacity is well established in most participating laboratories.

Qualitative concordance can mask quantitative interlaboratory bias. Analysis of Ct value distributions showed that several laboratories reported systematic differences in Ct values relative to the NCFAD reference values. These differences may be attributable to variations in analytical methods (e.g., reagents, master mixes, extraction kits, and protocols), instrumentation (e.g., thermocyclers and calibration status), and operator techniques (e.g., pipetting and master mix preparation). Such shifts are analytically important, as they may correspond to a substantial reduction in detectable template levels, potentially resulting in false-negative results.

Specific laboratory examples illustrate these general patterns. Lab 3, a regional reference laboratory, reported multiple elevated CSFV Ct values while using the Eberling et al., CSFV assay with the MagMAX 96 Total RNA extraction kit; the combination of elevated Ct values and assay choice implicates assay sensitivity or primer/probe mismatch to the panel strains rather than extraction performance alone. Lab 9, a food analysis laboratory, reported inconsistent CSFV real time PCR results, producing false negatives despite using an undisclosed automated magnetic bead kit; this pattern is consistent with extraction failure, PCR inhibition, or reporting omissions and highlights that automated platforms do not eliminate workflow or reporting errors. The isolated false-positive detection of S12, an ASFV sample, as CSFV by Lab 2 (Table 4), which was not detected by NCFAD or any other participating laboratory, raises additional concern and may indicate contamination, non-specific amplification, or a reporting error. Labs 2 and 6 used the same commercial kit for CSFV detection (Table 4); however, the Ct values they reported across all samples showed a notable difference, suggesting that the results were likely influenced by other factors, including the different extraction kits used by the two laboratories.

In contrast, the serological detection revealed greater performance variability, particularly for CSF. While 80% of laboratories achieved complete accuracy for ASF ELISA, only 35.7% did so for CSF ELISA, with 64.3% missing between 1 and 3 samples (Tables 5, 6). The differences in laboratory workflows and possibly the diversity of ELISA kits used in the PT likely contributed to the result variation. During result review at the NCFAD, a contributor to inconsistent ELISA results was the use of short incubation periods (1–2 h), rather than the overnight (~18 h) incubation specified in most kit manuals for better sensitivity. All the laboratories (except lab 20, IDEXX CSF Ab kit) that got accurate results for all CSF ELISA samples used the overnight sample incubation method. Reduced incubation time decreases assay sensitivity and increases the likelihood of borderline or false-negative results, particularly in samples with low antibody titers. The laboratories that utilized short sample incubation were encouraged to adopt overnight incubation, which should significantly improve their diagnostic performance. Samples 4 and 17 are expected to be positive in E2-based ELISA and negative in Erns-based ELISA, reflecting vaccine-induced antibodies against CSFV E2 but absence of antibodies to Erns, which are only produced after natural infection. This does not appear to impact the results submitted by laboratories, as those using E2-based ELISA still obtained negative results for these two samples in the absence of overnight sample incubation.

The stability of ASFV and CSFV nucleic acid in the presence of MTM enabled shipping the molecular PT panel at regular shipping temperatures without compromising integrity. This reduced the shipping cost paid by the participants and increased the accessibility of the NCFAD PT panels to laboratories in resource-limited countries. Some participating laboratories encountered shipping delays and customs clearance delays, which did not affect the results but slightly delayed reporting. These challenges are common in international PT programs and further highlight the need for enhanced coordination, early shipment planning, proper documentation, and clear communication channels between the providing and participating laboratories. Despite these challenges, all participating laboratories completed their PT panels and showed interest in strengthening their diagnostic capacity.

Several exercises on ASF or CSF PT/inter-laboratory comparison testing (ILCT) have been successfully conducted previously (31, 39–42), although they differed in scope, participating countries and laboratories, virus types, sample preparation methods, and panel distribution approaches. One of the key advantages of our PT exercise is that it incorporates both ASF and CSF testing, combining molecular and serological screening assays. Our study also validated the use of ambient shipping temperatures with MTM, thereby eliminating the need for cold-chain transport and reducing shipping costs. Additionally, our exercise focused on a diverse range of laboratory types across the Americas, with the aim of supporting capacity building in the region for rapid ASF and CSF detection and surveillance.

The molecular panel used in this exercise was prepared using inactivated cell culture-amplified ASFV and CSFV samples. The ideal sample types for preparing molecular panels are whole blood and tissue homogenate, which typically require cold chain for shipping. Recently, we have shown that whole blood, spleen swabs, and oral fluid samples could be shipped at ambient temperature after treatment with MTM for virus inactivation and viral nucleic acid preservation (22). This approach will be considered for preparing molecular panels in future PT exercises.

Differential diagnosis for ASF and CSF was neither requested nor expected in this exercise, as the primary objective was to ensure that participating laboratories in Central and Latin America were capable of performing real-time PCR and ELISA, the frontline high-throughput diagnostic assays used for ASF and CSF. These assays are essential for the rapid identification of suspected cases and for surveillance activities in these countries. Most participating laboratories did not have the resources required to perform confirmatory and/or differential diagnostic assays for ASF and CSF.

Following the conclusion of the exercise, detailed laboratory-specific and general reports were prepared and sent to each participating laboratory. Additionally, a virtual workshop was organized by the NCFAD to bring together representatives from the laboratories involved in the PT exercise. This provided participants with the opportunity to ask questions and share insights. In addition, the workshop was critical as it helped in fostering shared learning, clarifying ELISA and real-time PCR methodologies, and enabling collaboration among participating laboratories. Open discussions during the workshop allowed participants to understand the rationale behind result discrepancies and exchange guides on troubleshooting ELISA and PCR workflows. Continued engagement across laboratories is essential for building a broader diagnostic community capable of responding effectively to ASF and CSF threats.

While this PT exercise provided essential baseline information regarding the diagnostic capacities of the participating laboratories, it also opened doors for sustained capacity building. Future PT exercises could incorporate additional assays, such as virus isolation, sequencing, multiplex real-time PCR, or Neutralization Peroxidase-Linked Assay (NPLA), as needed, thereby expanding laboratory competency. Although we did not incorporate z-score in this round of PT, using it in future PT or ILCT exercises would allow laboratories to standardize their performance over time.

In conclusion, this NCFAD’s first ASF and CSF PT exercise targeting diagnostic laboratories in the Americas successfully assessed diagnostic capacity across a diverse group of laboratories and revealed detection capability alongside areas of improvement in ELISA and PCR. The PT exercise, in addition to the targeted feedback and a collaborative workshop, provided useful information that will assist laboratories in refining their diagnostic workflows and improving assay reliability. As the NCFAD plans to organize PT or ILCT exercises every two years, continued participation by laboratories is critical in enhancing regional preparedness, ensuring diagnostic accuracy, and contributing to global efforts to prevent and control ASF and CSF.

## Data Availability

The original data presented in the study are included in the article, further inquiries can be directed to the corresponding authors.
